# Impact of Weight Loss Due to Sleeve Gastrectomy on Shear Stress of the Femoral Vein in Morbid Obesity

**DOI:** 10.1007/s11695-013-1175-9

**Published:** 2014-01-14

**Authors:** Maciej Wiewiora, Jerzy Piecuch, Marek Glück, Ludmila Slowinska-Lozynska, Krystyn Sosada

**Affiliations:** 1 Department of General and Bariatric Surgery and Emergency Medicine in Zabrze, Medical University of Silesia, ul. Sklodowskiej-Curie10, 41-800 Zabrze, Poland; 2 Department of Biophysics, Medical University of Silesia, Zabrze, Poland

**Keywords:** Obesity, Venous wall shear stress, Sleeve gastrectomy

## Abstract

**Background:**

Studies have shown that obesity is associated with venous flow disturbances that lead to changes of the biomechanical forces on the venous wall known as shear stress. We hypothesized that weight loss due to bariatric surgery affects the venous hemodynamics and biomechanical forces on the venous wall. The aim of this study was to evaluate the effects of laparoscopic sleeve gastrectomy (LSG) on the wall shear stress (WSS) and the venous hemodynamics of the femoral vein.

**Methods:**

We studied ten morbidly obese patients who underwent LSG. We investigated venous hemodynamics before, 6 and 12 months after LSG. The femoral vein diameter, cross-sectional area, peak (PeakV) and maximum (TA_max_) velocities, WSS, and shear rate (SR) were assessed.

**Results:**

PeakV and TA_max_ were significantly lower in the obese patients compared with the control group. WSS and SR were significantly lower in the obese patients compared with the control subjects. Venous hemodynamic parameters increased in the postoperative period at baseline compared with 12 months after surgery: PeakV increased from 17.53 (14.25–20.01) cm/s to 25.1 (20.9–30.1) cm/s (*P* = 0.04) and the TA_max_ from 12.97 (11.51–14.6) cm/s to 18.46 (13.24–24.13) cm/s (*P* = 0.057). WSS significantly increased from 0.21 (0.19–0.23) Pa at baseline to 0.31 (0.23–0.52) Pa 12 months after surgery (*P* = 0.031). SR also significantly increased from 47.92 (43.93–58.55) s^−1^ at baseline to 76.81 (54.04–109.5) s^−1^ 12 months after surgery (*P* = 0.02).

**Conclusions:**

This study showed that weight loss due to LSG significantly changes the biomechanical forces on the femoral vein generated by blood flow.

## Introduction

The results of recent studies suggest that obesity is associated with an increased risk of venous disease, but the main mechanisms leading to the development of chronic venous disease are not clearly defined. The previous studies have shown that obesity is associated with an increase in both intraabdominal and iliofemoral venous pressure [1], greater foot venous pressure, and deep venous reflux [2]. Recent studies also suggest that inflammatory processes play an important role in determining vein wall damage, especially in the venous valve in patients with venous disease [3, 4].

These studies confirmed that wall shear stress is a product of the whole blood viscosity and the shear rate effect of endothelium function [5], the migration of leukocytes, coagulation, and inflammatory reactions [6]. These studies also suggest that shear stress may affect factors determining the risk of venous thrombosis and progressive grades of chronic venous insufficiency. Other authors have recently reported that obesity is associated with alteration of shear stress and venous hemodynamics in the lower limbs [7]. The present study has shown significantly lower shear stress at the femoral vein in the obese individuals compared with the control subjects. It is known that low shear stress and turbulent or reversed blood flow lead to inflammatory reactions, which has recently been recognized as an important etiologic factor of venous disease [8]. To our knowledge, the effects of weight loss due to bariatric surgery on wall shear stress of the femoral vein have not yet been established. The aim of this study was to evaluate the effects of weight loss due to sleeve gastrectomy on the wall shear stress of the femoral vein in morbid obesity.

## Material and Methods

The study included ten morbidly obese patients without signs and symptoms of chronic venous disease who underwent laparoscopic sleeve gastrectomy. Patients with varicosities, lower extremity edema, pigmentation or lipodermatosclerosis, and healed or active venous ulcers were excluded. Additional factors used as exclusion criteria included the following: smoking, diabetes mellitus, uncontrolled hypertension, heart disease, history of leg vein thrombosis, history of antithrombotic and/or estrogen and/or contraceptive therapy, clinical signs of arterial disease, prior limb surgery or sclerotherapy, and lack of patient consent to participate. The exclusion criteria related to using estrogen, contraceptive therapy, anticoagulants, and antiplatelet agents were applied to a period of 3 months before the study date. Ultrasound examinations excluded patients with evidence of a valve dysfunction or thrombosis. Anthropometric, hemodynamic, and biomechanical parameters before the operation were compared to those of the control group.

Patients enrolled into the study underwent laparoscopic sleeve gastrectomy (LSG). In this study, LSG was performed according to the commonly used technique [9, 10]. Greater curvature vessels were divided using the LigaSure device (Covidien™, USA). A longitudinal resection of the stomach was created using a linear cutting stapler (Echelon 60 Flex Endopath®, Ethicon Endosurgery, USA) beginning from the point 5 to 6 cm proximal to the pylorus and continuing to the angle of His, tightly abutting the bougie (a 36-French) that was placed transorally into the pyloric channel along the lesser curvature.

Patients were followed for 1 year following the procedure. The patients were weighed, examined, including a Doppler ultrasound exam, and interviewed by the surgeon and blood was drawn for whole blood viscosity measurements, preoperatively and 6 and 12 months after the operation.

The control group consisted of 18 nonobese people without arterial hypertension, diabetes mellitus, or any of the other above-listed features. This group was recruited from volunteers from the medical staff. The study protocol was approved by the ethical committee of the Medical University of Silesia, and all participants provided written consent for participation in the study.

Blood samples were collected from the cubital vein with a syringe for a biochemical examination and anticoagulated with K_3_EDTA (milligrams per milliliter) for rheological tests. The rheological tests were performed at a stable temperature of 37 °C within 2 h after the blood was collected. Whole blood viscosity measurements were performed using a Brookfield DV-II+ (Wells-Brookfield, USA) cone-plate viscometer at shear rates of 250 s^−1^.

Duplex ultrasound assessments of venous flow were performed with a LOGIQe (GE Healthcare, Wipro, India) scanner using an 8-MHz linear array probe. Ultrasound examinations were performed in a standardized supine position with an upper body elevation of 10° during normal quiet respiration. Venous flow dynamics and the vein diameter were measured at the common femoral vein within 1–1.5 cm proximal to the entrance of the greater saphenous vein. The flow velocity profile was studied in a longitudinal section of the common femoral vein, and a spectral Doppler image was obtained in 7–10 s from the center of the vessel. The vein’s diameter was measured by identifying a transverse scan on B-mode imaging of the intimal-luminal interfaces. The flow velocity profile was studied in a longitudinal section of the common femoral vein, obtaining a spectral Doppler signal. The femoral venous flow velocity was measured if the angle between the ultrasound beam and the longitudinal vessel axis was 60° or less. Dedicated software provided enveloping of the spectral waveforms with automated estimation of the Peak velocity (PeakV) and time-averaged maximum velocity (TA_max_). After 1 min for stabilization, PeakV, TA_max_, and vascular diameter were obtained. Three measurements were performed for each of the evaluated parameters at 1 min intervals, and the mean value of each measurement was used for the final calculations. Ultrasound settings, such as gain, contrast, and rejection, were optimized during the initial evaluation and held constant thereafter. The cross-sectional area of the femoral vein was calculated from the formula *π* × D^2^/4, where D denotes diameter. Shear rate was estimated from the measured blood flow velocity and the diameter of the femoral vein according to the equation: *γ* = 4 × V_max_/D [11], where V_max_ denotes maximal velocity, which in our study corresponds to TA_max_. We applied the equation that is used for estimating the flow in the arteriole. The venous wall shear stress (*τ*) was calculated from the blood viscosity (*η*) and the shear rate (γ) assuming Newton’s law. Wall shear stress was calculated from the whole blood viscosity at shear rates of 250 s^−1^. At this shear rate, blood may be regarded as a Newtonian fluid [12]. Validation and reproducibility of this method for estimating shear stress have been described previously [13].

### Statistical Analysis

The continuous variables are presented as the median and the interquartile range. The categorical variables are presented as the absolute numbers and percentages. Statistical comparisons were achieved by using the Mann–Whitney *U* test for independent groups: morbidly obese vs. control groups prior to surgery and morbidly obese vs. control groups 12 months after surgery. Fisher’s exact test was performed to compare differences for categorical data. Differences in each variable at the three time points (before surgery and 6 and 12 months after surgery) were calculated by the Friedman ANOVA test followed by Scheffe’s post hoc test. A *P* value <0.05 was considered significant. The statistical analysis was performed using Statistica 10 (StatSoft Inc.).

## Results

**Fig. 1:**
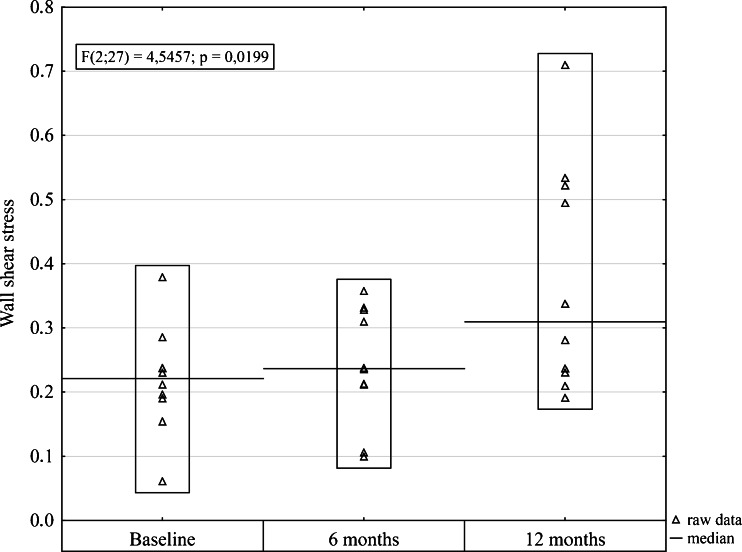
Box plot comparing wall shear stress at each time point before and after surgery

**Fig. 2:**
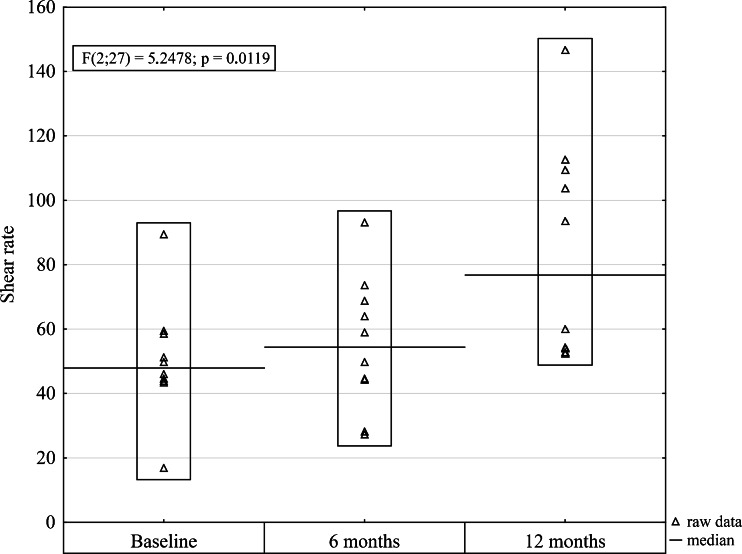
Box plot comparing shear rate at each time point before and after surgery

The baseline characteristics of the study population and their comparisons to the control group are presented in Table 1. The gender distribution and ages were similar in the obese and control groups. Anthropometric parameters were respectively higher in the obese subjects. The femoral vein diameter and the cross-sectional area were significantly higher in the obese group than in the control group (Table 1). PeakV and TA_max_ were significantly lower in the obese patients compared with the control group (Table 1). The shear rate and the wall shear stress were significantly lower in the morbidly obese group than in the control group. Anthropometric parameters changed at each time point after surgery. The median weight and BMI significantly decreased at each time point after surgery compared to the preoperative value (Table 2). WHR, diameter, and cross-sectional area of the vein significantly decreased 12 months after the operation compared to the baseline value (Table 2). Venous hemodynamic parameters increased postoperatively at each time point. Significant differences were observed for PeakV, which elevated from 17.53 (14.25–20.01) cm/s at baseline compared to 25.1 (20.9–30.1) cm/s 12 months after surgery (*P* = 0.040, Table 2); TA_max_ tended to be higher at baseline compared to 12 months after surgery from 12.97 (11.51–14.6) cm/s to 18.46 (13.24–24.13) cm/s (*P* = 0.057, Table 2). The same increasing trend was observed for shear rate and wall shear stress. Wall shear stress significantly increased from 0.21 (0.19–0.23) Pa at baseline to 0.31 (0.23–0.52) Pa 12 months after surgery (*P* = 0.031; Table 2 and Fig. 1). Shear rate also significantly increased from 47.92 (43.93–58.55) s^−1^ at baseline to 76.81 (54.04–109.5) s^−1^ 12 months after surgery (*P* = 0.020; Table 2 and Fig. 2). There were no differences in the venous hemodynamics of the lower limbs between the obese and control participants 12 months after surgery (Table 3).

**Table 1:** Baseline characteristics of obese patients and control participants

		Morbid obesity *N* = 10	Control *N* = 18	*P* value
Gender	Male	4 (40 %)	4 (22.2 %)	0.369
	Female	6 (60 %)	14 (77.8 %)	0.461
Age (years)		38.5 (33–40)	37 (27–40)	0.615
Weight (kg)		150.5 (126.5–160)	67 (62–73)	<0.0001
BMI (kg/m^2^)		49.2 (44.1–52.9)	23.85 (22.45–24.65)	<0.0001
WHR		1.09 (0.97–1.14)	0.8 (0.78–0.85)	<0.0001
PeakV (cm/s)		17.53 (14.25–20.01)	24.92 (17.15–31.9)	0.044
TA_max_ (cm/s)		12.97 (11.51–14.6)	16.15 (13.2–21.7)	0.046
D (mm)		10.8 (10–11.4)	8.75 (8–9.2)	<0.001
CSA (cm^2^)		0.91 (0.78–1.02)	0.6 (0.5–0.66)	<0.001
Wall shear stress (Pa)		0.21 (0.19–0.23)	0.31 (0.22–0.39)	0.032
Shear rate (s^−1^)		47.92 (43.93–58.55)	76.89 (60.92–99.34)	0.0023
Blood viscosity at 250 s^−1^ (mPa s)		4.38 (3.71–4.77)	3.88 (3.66–4.15)	0.041

Data are presented as the medians (interquartile ranges) for continuous variables or the prevalences for categorical data. Group differences were calculated using the unpaired Mann–Whitney *U* test for non-normal distribution data and the Fisher’s exact test for categorical data

*BMI* body mass index, *WHR* waist to hip circumference ratio, *PeakV* peak velocity, *TA*_*max*_ time-averaged maximum velocity, *D* diameter, *CSA* cross-sectional area of the vein

**Table 2:** Anthropometric parameters and venous hemodynamics of the lower limbs at each time point before and after surgery

	Baseline *N* = 10	6 months *N* = 10	12 months *N* = 10	*P* value	
				6 months vs. baseline	12 months vs. baseline
Weight (kg)	150.5 (126.5–160)	105.5 (102–126)	100 (97–108)	0.016	0.00034
BMI (kg/m^2^)	49.2 (44.1–52.9)	39.5 (34.2–40.7)	34.8 (31.3–37)	0.010	0.0001
WHR	1.09 (0.97–1.14)	0.95 (0.92–1.08)	0.9 (0.88–0.96)	0.127	0.0068
PeakV (cm/s)	17.53 (14.25–20.01)	17.14 (14.8–22.71)	25.1 (20.9–30.1)	0.979	0.040
TA_max_ (cm/s)	12.97 (11.51–14.6)	13.44 (11.18–16.21)	18.46 (13.24–24.13)	0.999	0.057
D (mm)	10.9 (10–11.4)	10 (8.8–11.5)	9.3 (8.2–9.8)	0.655	0.036
CSA (cm^2^)	0.9 (0.78–1.02)	0.79 (0.6–1.07)	0.68 (0.52–0.75)	0.702	0.039
Wall shear stress (Pa)	0.21 (0.19–0.23)	0.23 (0.21–0.32)	0.31 (0.23–0.52)	0.906	0.031
Shear rate (s^−1^)	47.92 (43.93–58.55)	54.4 (44.27–68.83)	76.81 (54.04–109.5)	0.907	0.020
Blood viscosity at 250 s^−1^ (mPa s)	4.38 (3.71–4.77)	4.62 (3.84–4.77)	4.62 (3.82–4.73)	0.952	0.952

Data are presented as median and interquartile range. Group differences were calculated by using the ANOVA test. For abbreviations, see Table 1

**Table 3:** Differences in the venous hemodynamics of the lower limbs of obese patients 12 months after surgery and control participants

	12 months *N* = 10	Control *N* = 18	*P* value
PeakV (cm/s)	25.1 (20.9–30.1)	24.92 (17.15–31.9)	0.942
TA_max_ (cm/s)	18.46 (13.24–24.13)	16.15 (13.2–21.7)	0.648
D (mm)	9.3 (8.2–9.8)	8.75 (8–9.2)	0.130
CSA (cm^2^)	0.68 (0.52–0.75)	0.6 (0.5–0.66)	0.119
Wall shear stress (Pa)	0.31 (0.23–0.52)	0.31 (0.22–0.39)	0.614
Shear rate (s^−1^)	76.81 (54.04–109.5)	76.89 (60.92–99.34)	0.980

Data are presented as the medians and interquartile ranges. Group differences were calculated using the unpaired Mann–Whitney *U* test. For abbreviations, see Table 1

## Discussion

Many obese patients have clinical signs of severe venous disease, including venous stasis ulcers and changes in the skin and subcutaneous tissue, such as pigmentation or lipodermatosclerosis related to venous stasis [14]. Epidemiological studies have demonstrated that obesity is associated with an increased risk of venous disease [15, 16], but the primary mechanisms leading to the development of chronic venous disease are not clearly defined. van Rij et al. found a higher incidence of severe venous disease in obese persons, according to clinical-etiologic-anatomic-pathophysiologic grading and greater foot venous pressure and reflux measures, such as the venous filling index and the venous filling time [2]. Another study demonstrated that obesity is associated with increased intra-abdominal and iliofemoral venous pressure that may contribute to the development of venous insufficiency in the lower limbs [1].

Chronic venous disease etiology has not been established yet and in its development many factors are taken into consideration, including genetic predisposition, lifestyle, physiological characteristics, and some comorbidities or hormonal therapy and contraception. Our study included obese patients without signs of venous disease with a well-controlled blood pressure. The factors used as exclusion criteria included those that could potentially influence the observed biomechanical parameters and blood viscosity. This allowed the creation of a homogeneous group of patients, minimizing the impact of hormonal factors and other pathologies associated with obesity on the measured biomechanical parameters.

The results of our study showed that obesity is associated with venous flow disturbances, which lead to changes of the biomechanical forces on the venous wall. The femoral vein diameter was significantly greater in obese participants compared with nonobese participants. Venous hemodynamic properties were lower in morbidly obese subjects. Lower velocities and greater diameter resulted in lower shear rates and wall shear stress. We have previously evaluated hemodynamic parameters and hemorheological parameters, including erythrocyte aggregation, among 35 obese patients qualified for bariatric surgery [17]. This study has found that shear rate and wall shear stress in obese subjects were lower than in the nonobese. The results of our study confirm those of other authors with regard to the same declining trend for shear stress and venous hemodynamics [7].

These hemodynamic changes in morbidly obese individuals are most likely related to the increased intra-abdominal pressure caused by abdominal fat [18, 19]. Willenberg et al. demonstrated that the application of external pressure to the abdomen, simulating the effects of abdominal obesity, impacts the venous flow and diameter of the femoral vein in a manner similar to that observed in obese patients [20].

The results of this and other studies indicate that obesity has a relevant impact on the biomechanical behavior of the venous wall of the lower limbs. Our data show a significantly lower value of shear stress at the femoral vein in morbidly obese individuals compared to nonobese participants. Lower shear stress might promote factors that support development of venous pathologies, as was described by Bergan et al. [8].

The vascular endothelium is exposed to a spectrum of fluid mechanical forces generated by blood flow; some of these, such as fluid shear stress, can directly modulate endothelial gene expression [21]. A previous study confirmed that wall shear stress is an important determinant of endothelial cell function [22]. Another study suggested that wall tension leads to the expression of many proteins, including matrix metalloproteinase in the venous wall [23]. Laminar shear stress is known to confer potent anti-inflammatory, antithrombotic, and anti-adhesive effects by differentially regulating endothelial gene expression [24–26].

The pathophysiology of venous disease depends on a constellation of interrelated risk factors. Inflammation has recently been recognized as an important etiologic factor for venous disease. Factors that influence the inflammatory process and structural remodeling in venous valves and in the venous wall remain unknown. On the other hand, it has been well documented that low shear stress and turbulent or reverse blood flow lead to thrombosis and inflammatory reaction, which has recently been recognized as an important etiologic factor for venous disease [6, 27, 28]. This raises the question: Can the weight loss due to bariatric surgery improve hemodynamic and biomechanical conditions of the lower limbs in obese patients? To our knowledge, the effects of weight loss due to bariatric surgery on biomechanical behavior of the venous wall of the lower limbs have not yet been established.

In this study, we observed a reduction in the diameter and cross-sectional area of the vein and an increase in the PeakV and maximal venous flow postoperatively after bariatric weight loss surgery. The increasing trend was observed for the biomechanical behavior of the venous wall of the lower limbs at each time point after surgery. The wall shear stress and the shear rate significantly increased 12 months after surgery. The differences in the venous hemodynamics of the lower limbs between the obese and control participants disappeared 1 year after surgery. The study was conducted in a group of ten obese patients who underwent sleeve gastrectomy. Measurements of hemodynamic parameters were performed before surgery and 6 and 12 months after surgery. All patients were subjected to a prospective postoperative control, according to a homogeneous, standardized research model. The study group was small, but a thorough analysis of the original data, presented in Figs. 1 and 2, clearly indicates that in all obese patients 12 months after surgery, there was a unidirectional increase in shear rate and wall shear stress.

Low shear stress promotes the migration of leukocytes, coagulation and smooth cell proliferation, and induces changes in endothelial cell function [6, 29, 30]. This effect of shear stress on pathological alterations may lead to the preservation and progression of venous dysfunction. Long-term adaptation in obese subjects can involve structural and functional remodeling of the venous wall. On the other hand, the beneficial effects of weight loss due to sleeve gastrectomy on the hemodynamics of blood flow and shear stress may cause the opposite effect.

The frequency of the occurrence of obesity in combination with lower extremity venous disease indicates a multifactorial relationship that, due to the excess weight, should also include biomechanical factors. This study showed that weight loss due to LSG significantly improves the biomechanical forces of the femoral vein generated by blood flow. Obviously, it cannot be concluded on the basis of the presented results that wall shear stress changes after weight loss due to bariatric surgery influence the occurrence and progression of venous disease in the obese. Further studies are needed to compare patients in relation to the grade of venous insufficiency symptoms.

## Study Limitations

The present manuscript was conceived as an initial prospective study assessing the effects of weight loss due to bariatric surgery on biomechanical behavior of the venous wall in the lower limbs. There are several limitations to the study that need to be acknowledged. Its results cannot be extrapolated to a general obese population because of the relatively small number of patients. The findings with respect to long-term biomechanical changes after bariatric surgery require larger prospective studies to confirm our results. In addition, we did not examine independent variables associated with postoperative weight loss which can affect the hemodynamics of flow and biomechanical behavior of the venous wall. The impact of exercise and activity on changes of the biomechanical forces on the venous wall before and after bariatric surgery might be interesting. There are, however, obvious methodological difficulties in directly assessing the effect of increased activity and exercise after surgery on biomechanical changes in the venous wall. This exploration would require a homogenous research model in which, in addition to surgery, there would be implemented a uniform set of exercises.
